# Microvillus inclusion disease-causing MYO5B point mutations exert differential effects on motor function

**DOI:** 10.1016/j.jbc.2025.108328

**Published:** 2025-02-18

**Authors:** Deanna M. Bowman, Leslie M. Meenderink, Kyra S. Thomas, Elizabeth H. Manning, Matthew J. Tyska, James R. Goldenring

**Affiliations:** 1 Department of Cell and Developmental Biology, Vanderbilt University, Nashville, Tennessee, USA; 2 Epithelial Biology Center, Vanderbilt University Medical Center, Nashville, Tennessee, USA; 3 Division of Infectious Disease, Department of Medicine, Vanderbilt University Medical Center, Nashville, Tennessee, USA; 4 Veterans Affairs Tennessee Valley Health Care System, Nashville, Tennessee, USA; 5 Section of Surgical Science, Vanderbilt University Medical Center, Nashville, Tennessee, USA

**Keywords:** actin, confocal microscopy, congenital diarrhea, intestinal epithelium, intracellular trafficking, microvilli, myosin

## Abstract

Microvillus inclusion disease (MVID) is a rare congenital diarrheal disorder typically caused by loss of function mutations in the unconventional myosin, myosin 5b (MYO5B), which leads to the mistrafficking of apical components in enterocytes. MVID can manifest in two phenotypes: in both the intestine and liver or the liver alone. Although previous studies seeking to understand MVID disease pathology used MYO5B KO models, many patients have point mutations and thus express a dysfunctional MYO5B. How these point mutations lead to a broad spectrum of disease severity and the development of two distinct disease phenotypes is still not known. Here, we investigate the effect of MVID patient mutations on the function of the MYO5B motor domain, independent of cargo binding, using confocal imaging and fluorescence recovery after photobleaching. Patient mutations demonstrated a range of effects in these assays, from rigor-like behavior to loss of actin binding. Additionally, analysis of fluorescence recovery after photobleaching turnover kinetics suggests that some mutations negatively impact the ability of MYO5B to stay bound to actin. Collectively, our findings indicate that patient mutations affect the MYO5B motor domain in diverse ways, consistent with the spectrum of phenotypes observed in patients.

Microvillus inclusion disease (MVID) is a rare congenital disorder that causes life-threatening secretory diarrhea and progressive familial intrahepatic cholestasis type 6 (1, 2, 3). MVID becomes apparent within the first few hours to days of life, as infants have watery diarrhea, dehydration, malnutrition, metabolic acidosis, and rapid weight loss (4, 5, 6, 7, 8, 9). A combination of examination of a small intestine biopsy and genome sequencing is used to diagnose MVID. Enterocytes in MVID patients show a loss of organization and packing of brush border microvilli, accumulation of tubulovesicular membranes and lysosomes in the apical cytoplasm, and large inclusions containing microvilli, other apical components, and lumen contents (10, 11). Other observations in MVID are abnormal subapical staining of CD10, villus blunting, crypt extension, and decreased tuft cell populations (12, 13, 14). Patients diagnosed with MVID require lifetime total parenteral nutrition to manage symptoms or a total intestinal and liver transplant to definitively treat the disease (15). With either option, patients with MVID face a lifetime of invasive, taxing, and expensive medical interventions. In addition to intestinal disease, patients develop progressive familial intrahepatic cholestasis type 6 over time, which is not due to the total parenteral nutrition (1, 16). In 2008, it was determined that mutations in the unconventional myosin, myosin 5b (MYO5B), can lead to MVID (17, 18). Interestingly, certain MYO5B mutations lead to cholestasis without causing a diarrhea phenotype (19, 20, 21). The existence of two different disease states poses the question of how mutations in the same protein can lead to a disease that affects both the intestine and liver or only the liver. In addition to a dichotomy in disease phenotype, a spectrum of disease severity is also seen in patients (21, 22). These factors add more complexity to understanding how MVID develops and establishing better treatment options for patients.

MYO5B is part of the myosin superfamily, actin-based motor proteins that convert chemical energy from ATP into mechanical work (23). The three unconventional mammalian class 5 myosins are MYO5A, MYO5B, and MYO5C, which have been implicated in subcellular functions ranging from cell motility, endocytosis, vesicle trafficking, and protein localization (24, 25, 26, 27, 28). Myosin-5 contains an N-terminal motor domain with ATPase and actin-binding activity, a central neck domain with six IQ motifs that bind calmodulin, a coiled-coil domain that drives dimerization, and a C-terminal globular tail domain that binds molecular cargos (29, 30, 31, 32, 33, 34, 35, 36). It is thought that MYO5B is a processive motor with a high duty ratio based on studies on MYO5B and the homolog chicken myosin-5 (37, 38, 39). Previous work indicated that MYO5B is critical for the apical delivery and recycling of transporters and enzymes in intestinal enterocytes (18, 28). MYO5B interacts with Rabs, which in turn allows interactions with specific vesicle populations. Rab proteins are small guanosine triphosphatases that act to aid in the delivery of cargo to the correct cell compartments. MYO5B interacts with RAB8A, RAB11A, RAB11B, RAB25, and RAB6A through its globular tail and also interacts with RAB10 through an alternatively spliced exon D sequence (28, 40, 41, 42, 43, 44). MYO5B can also form a ternary complex with RAB11A and RAB11-FIP2 (45, 46). In MVID, MYO5B dysfunction leads to the mislocalization of numerous apical components, including sodium-glucose cotransporter 1, aquaporin7, CDC42, sucrase-isomaltase, alkaline phosphatase, sodium-hydrogen exchanger 3, phosphoinositide-dependent protein kinase 1, and cyclic guanosine monophosphate–dependent protein kinase, but cystic fibrosis transmembrane regulator protein is retained at the apical surface (47, 48, 49). We previously noted that the MYO5B(P660L) mutation results in diffuse cytoplasmic staining of Rab11a in patient samples, but the MYO5B(G519R) patient mutation induces subapical accumulation of Rab11a with MYO5B in enterocytes (47, 50). The difference in Rab11a localization in patient samples suggests that the inactivating mutations differentially impact the motor and may affect intracellular trafficking and cell biology in different manners.

There are currently no approved drug treatments to reduce the severity of diarrhea in MVID patients or bypass deficits in MYO5B-dependent apical trafficking. Recent work in mouse models has shown that lysophosphatidic acid can reduce villus blunting, inhibit large lysosome formation, increase apical sodium-glucose cotransporter 1 activity, decrease cystic fibrosis transmembrane regulator activity, and increase tuft cell populations (51, 52). Previous studies in cell and animal models primarily used complete genetic knockout of MYO5B to model MVID. Yet many MVID-causing mutations are missense mutations and can be compound heterozygous (11, 53, 54, 55, 56, 57, 58). Recent studies used mice to model the patient compound heterozygous mutation, MYO5B(G519R), with a second early termination mutation (50). That work demonstrated the need for more patient-driven models as an effective method to study MVID and also highlighted that effective treatment of MVID may differ depending on the patient mutation. Unlike other genetic disorders caused by missense mutations, mutant MYO5B is still expressed in MVID patients. We sought to evaluate how specific point mutations affect the MYO5B motor, with the goal of developing insights into MYO5B-dependent apical trafficking.

In this study, we took optimized a new cell-based assay to investigate the functional impact of MVID-causing point mutations on MYO5B motor activity. Given the spectrum of disease phenotypes observed in patients with MYO5B mutations, our goal was to determine if MVID point mutations manifested unique or categorizable phenotypes. Our assay allowed us to examine motor function independent of the cargo binding and enabled us to focus on defining the impact of disease-causing mutations in live cells. Using this approach, we discovered that defined mutations with a known outcome and patient-derived, disease-causing mutations impacted motor function and dynamics. The effects of the patient-derived mutations reported here will inform how a spectrum of disease is formed from single MYO5B point mutations in MVID patients and can impact the direction of treatment.

## Results

### Development of an in-cell assay for MYO5B motor function

**Figure 1:**
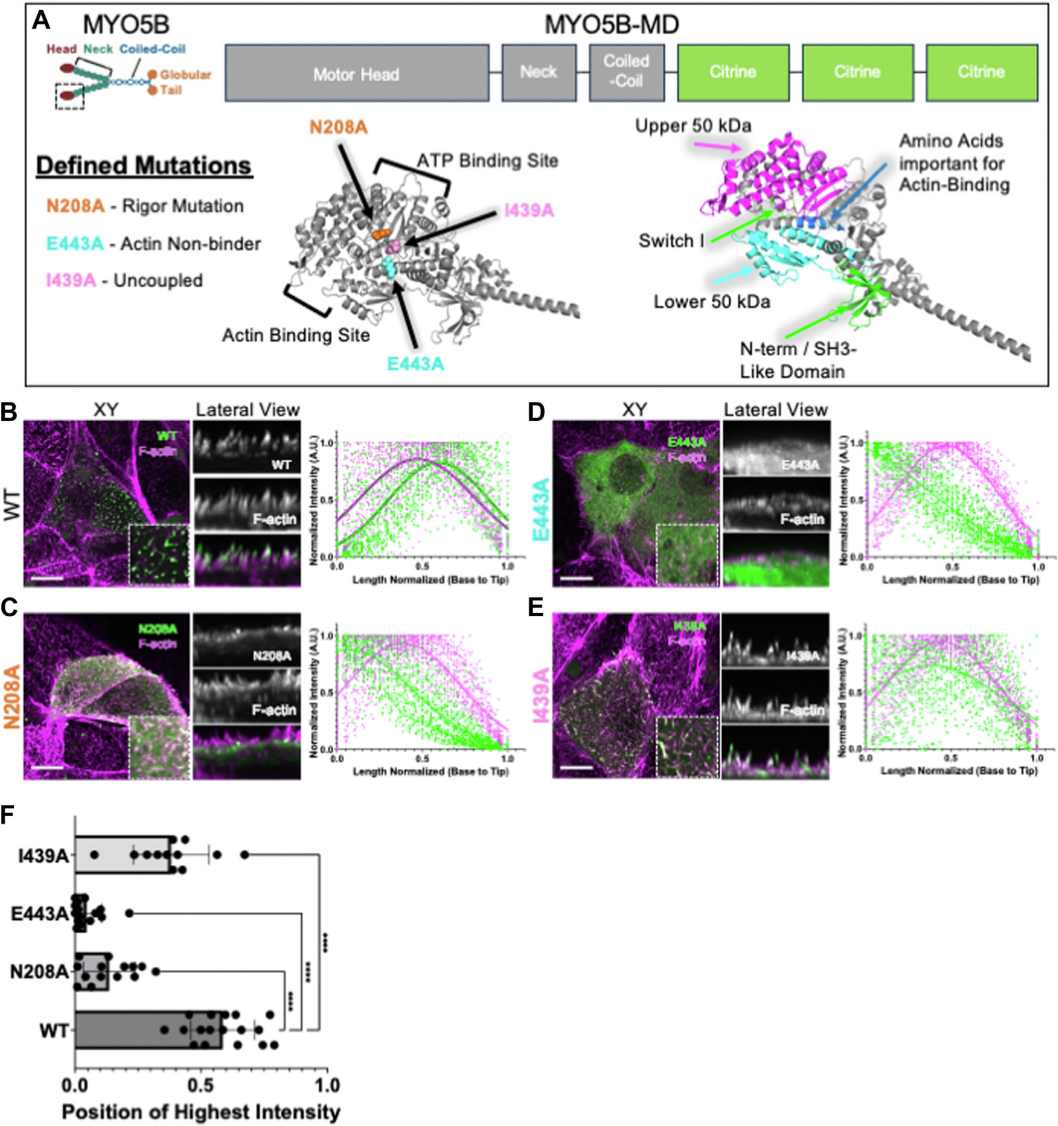
**Point mutations in the MYO5B motor with a predicted outcome disrupt normal MYO5B motor localization**. *A,* MYO5B dimer diagram (*top left*), *boxed area* shown below using an AlphaFold structure of the MYO5B motor head indicating positions of prediction point mutations (*left*) and important actin-binding domains (*right*). MYO5B-MD expression construct diagram. *B,* WT MYO5B-MD constructs are more localized to the tips of microvilli. *C,* predicted rigor mutation, N208A, localizes to the bases of microvilli. *D,* predicted actin-nonbinding mutation, E443A, localizes to the cytoplasm of the cells. *E,* predicted uncoupled mutation, I439A, localizes along the length of microvilli. *F,* the point along the normalized length of microvilli at which the intensity of the MYO5B-MD-CC-3x-Citrine signal was the highest plotted. ∗∗∗∗*p* value <0.0001. Each condition has an N ≥ 12 cells, where five or more microvilli have been measured from a replicate of three independent coverslips. Phalloidin is used to mark F-actin (*magenta*), and the MYO5B motor was transiently transfected into the cells (*green*). The scale bar represents 10 μm. MYO5B, myosin 5b.

To investigate the effect of specific patient-derived MVID-causing mutations on MYO5B motor function, we sought to develop an assay that would enable us to circumvent laborious protein purification and single-molecule assays that are typically used to examine myosin motor function *in vitro*. We based our approach on the LLC-PK1-CL4 (CL4) line, kidney epithelial cells that build apical microvilli containing unipolar actin bundles, which we reasoned should support directed motility by processive myosins such as MYO5B. To test this idea, we transfected CL4 cells with a chimeric construct of MYO5B (a.a. 1-1015)-3x-Citrine, which contained the motor domain, neck domains, and the proximal coiled-coil domain linked to a C-terminal 3X-Citrine sequence (MYO5B-MD). This construct is expected to dimerize, but not interact with known binding partners given the lack of the globular tail domain. Inspection of CL4 cells expressing MYO5B-MD revealed striking enrichment at the distal tips of apical microvilli (Fig. 1*B*). For previously characterized processive myosins studied in the context of microvilli, stereocilia, and filopodia, tip accumulation serves as a telltale sign of barbed end–directed motor activity (59, 60). Therefore, we quantified the localization of WT MYO5B-MD (Fig. 1*A*) signal relative to the phalloidin signal (used to mark F-actin in microvilli) by generating line scans along the base-to-tip axis of individual microvilli, with the resulting barbed end enrichment of signal as a readout for motor activity.

### Calibration of the in-cell motor assay using established loss-of-function mutations

To calibrate our in-cell motor assay, we created MYO5B-MD constructs that contained previously characterized mutations in highly or perfectly conserved residues of the myosin motor domain (Fig. 1*B*) (61, 62, 63, 64, 65). These mutations fall into three classes with regard to impact on motor domain activity: (1) N208A, a mutation that prevents ATP binding, locking the motor in a strong actin-bound state (“rigor”); (2) E443A, a mutation that blocks phosphate release, inhibiting actin binding (“nonbinder”); and (3) I439A, a mutation in the switch II region that uncouples ATP hydrolysis from the power stroke resulting in a loss of force production (“uncoupled”) (Fig. 1*A*). The location of these mutations in a sequence alignment is shown in Fig. S1. These three classes of mutations were used as defined points of comparison for patient mutations. To examine their impact on MYO5B motor domain function in cells, mutant variants of MYO5B-MD were expressed in the LLC-PK1-CL4 cells, and their localization along the microvillar axis was examined (Fig. 1, *C*–*E*). A MYO5B-MD WT construct demonstrated strong accumulation at the tips of microvilli, reflecting barbed end–directed motor activity. A Gaussian fit to the F-actin signal peaked at 0.47 ± 0.0048, while the peak for the WT MYO5B motor was more distal at 0.60 ± 0.0048 (Fig. 1*B*). In contrast, a MYO5B construct with the predicted rigor mutation, MYO5B-MD(N208A), lacked tip enrichment and localized basal to the F-actin peak of (0.07 ± 0.0135 *versus* 0.39 ± 0.003, respectively) (Fig. 1*C*), indicating impaired motility. A MYO5B construct expected to lack strong actin binding, MYO5B-MD(E443A), demonstrated dispersed cytosolic localization, resulting in an unfittable intensity profile (Fig. 1*D*). A mutant containing a predicted uncoupling mutation, MYO5B-MD(I439A), was still able to enter microvilli, but exhibited peak localization that was basal to the peak F-actin signal (0.39 ± 0.0095 *versus* 0.44 ± 0.0033, respectively) (Fig. 1*E*), again indicating impaired tip-directed motility.

For each of these mutant MYO5B motors, we examined the position along the normalized microvillus length axis that had the highest intensity, which served as points of comparison between constructs. Each of the predicted conserved motor domain mutation sites resulted in a statistically significant change in the location of the peak along the microvillus axis (Fig. 1*F*). Together, these results provided a framework for interpreting the impact of uncharacterized patient mutations on MYO5B motor activity in the in-cell motor assay.

### MVID-causing MYO5B mutations impair MYO5B motor function

**Figure 2:**
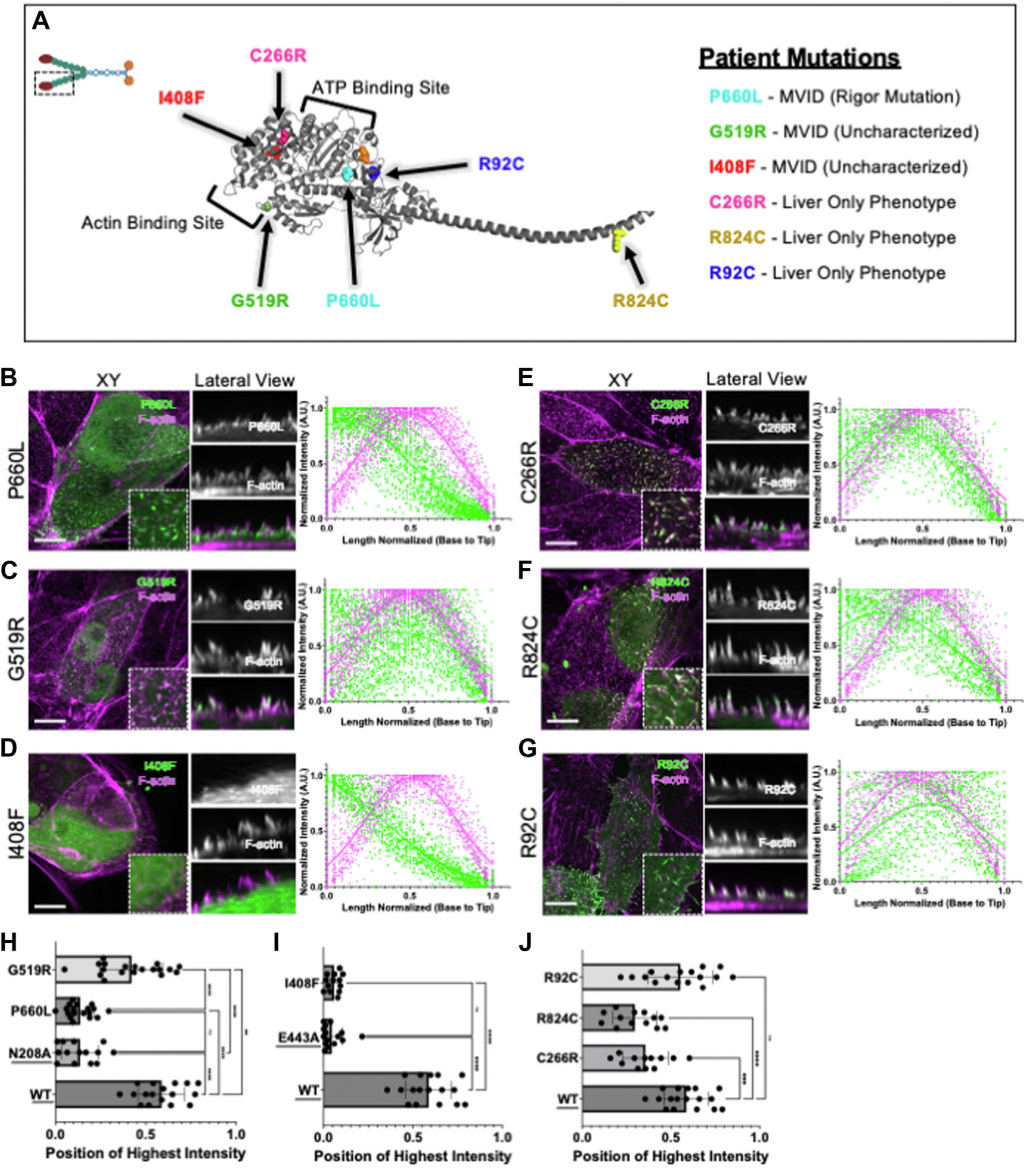
**Patient MYO5B motor mutations disrupt normal MYO5B localization.***A,* position of patient mutations shown on an AlphaFold structure of MYO5B motor head. *B,* patient mutation, P660L, localizes to the bases of microvilli. *C,* patient mutation, G519R, localizes along the length of microvilli. *D,* patient mutation, I408F, localizes intracellularly. *E,* patient mutation, C266R, localizes along the length of the microvilli. *F,* patient mutation, R824C, localizes more toward the bases of microvilli. *G,* patient mutation, R92C, localizes along the length of microvilli and more toward the tips of microvilli. *H,* the point along the normalized length of microvilli at which the intensity of the MYO5B-MD signal was the highest plotted for WT MYO5B-MD, MYO5B-MD(N208A), MYO5B-MD(P660L), and MYO5B-MD(G159R). *Underlined mutants* were shown in the previous figure. *I,* the point along the normalized length of microvilli at which the intensity of the MYO5B-MD signal was the highest plotted of the WT MYO5B-MD, MYO5B-MD(E443A), and MYO5B-MD(I408F). *Underlined mutants* were shown in the previous figure. *J,* the point along the normalized length of microvilli at which the intensity of the MYO5B-MD signal was the highest plotted of the WT MYO5B-MD, MYO5B-MD(C266R), MYO5B-MD(R824C), and MYO5B-MD(R92C). *Underlined mutants* were shown in the previous figure. ∗∗∗∗*p* value <0.0001. Each condition has an N ≥ 12 cells, where five or more microvilli have been measured from a replicate of three independent coverslips. Phalloidin is used to mark F-actin (*magenta*), and the MYO5B motor was transiently transfected into the cells (*green*). The scale bar represents 10 μm. MYOB, myosin 5b.

Patient mutations analyzed in our experiments were published previously (16, 17, 20, 50, 66) or shared with us by collaborators (Fig. 2*A*), representing two classes of disease phenotypes: (1) mutations that cause intestinal and liver disease (P660L, G519R, and I408F) and (2) mutations that only cause liver disease (C266R, R824C, and R92C). The mutations are located throughout the MYO5B motor domain (Figs. 1*A* and S1). The P660L mutation is located within a region which is important for actin binding. The G519R mutation is located within the lower 50 kDa of the motor domain (35). The I408F and C266R mutations are located within the upper 50 kDa of the motor domain (35). Both upper and lower 50 kDa domains are implicated in actin binding. The R824C mutation is located within the second IQ2 domain, which binds calmodulin and is part of the neck domain (35). The R92C mutation is located in the N-terminal domain within the motor domain (35). Our laboratory previously described the P660L mutant as a potential rigor mutation, and the location within the actin binding cleft could further support a rigor mutation (47, 50). Patient phenotype, outcomes, and biopsy staining have primarily determined the association of these patient mutations with MVID. The link between the location of the patient mutation within the MYO5B protein with disease phenotype and outcome is currently not understood; mutations resulting in both intestinal and liver phenotypes are found throughout the MYO5B motor protein (67). These mutations are also in conserved areas (Fig. S1). Our laboratory previously described the Navajo P660L mutation as a potential rigor mutation (47, 50). In human symptomatic MVID patients, mutations P660L, I408F, C266R, and R824C are homozygous. The patient with the G519R had a compound heterozygous mutation, with the G519R mutation on one allele and an early truncation mutation leading to an early termination on the other allele (50). The liver-specific patient with the R92C mutation had a compound heterozygous mutation with a R92C mutation on one allele and a H138P mutation on the other allele.

We first examined the patient mutations resulting in intestinal and liver disease. Mutant MYO5B-MD(P660L) primarily localized at the bases of microvilli resulting in the peak of MYO5B-MD(P660L) to be basal to the F-actin excitation peak (0.11 ± 0.0092 and 0.48 ± 0.0023, respectively) (Fig. 2*B*). The localization of MYO5B-MD(P660L) resulted in a shift in localization of the MYO5B-MD construct, similar to the predicted rigor mutant, MYO5B-MD(N208A). MYO5B-MD(G519R) was able to enter microvilli and localized along the length of the microvilli and exhibited peak excitation slightly basal to the peak of F-actin excitation (0.40 ± 0.0109 and 0.49 ± 0.0020, respectively) (Fig. 2*C*). This could suggest that the motor is slower or less efficient than WT MYO5B-MD. The uncharacterized patient variant MYO5B-MD(I408F) localized diffusely in the cytoplasm, resulting in an unfittable intensity profile (Fig. 2*D*). The localization of MYO5B-MD(I408F) was similar to that for the predicted actin nonbinding motor, MYO5B-MD(E443A).

Next, we evaluated the axial position of highest intensity as a measure of motor function. MYO5B-MD(G519R) showed a significant decrease in localization intensity along microvilli (Fig. 2*H*). We then also compared the WT MYO5B-MD with the predicted mutant causing a rigor mutation (N208A) and the patient P660L mutation. There was a marked and significant decrease in the localization of the point of highest motor intensity closer to the bases of microvilli in both the MYO5B-MD(N208A) and MYO5B-MD(P660L) mutations when compared to the WT motor (Fig. 2*H*). No significant difference was noted between the MYO5B-MD(N208A) and the MYO5B-MD(P660L). The predicted actin nonbinding mutation (E443A) was compared to the patient mutation MYO5B-MD(I408F) which showed similar localization. There was a significant decrease in both MYO5B-MD(E443A) and MYO5B-MD(I408F) when compared to WT MYO5B-MD, but there was no significant difference between MYO5B-MD (E443A) and MYO5B-MD(I408F) (Fig. 2*I*).

We also examined the impact of the liver-specific MYO5B mutants on the localization of the MYO5B motor in this assay. MYO5B-MD(C266R) localized along the length of microvilli, with a peak signal that was basal to the F-actin peak (0.33 ± 0.0051 *versus* 0.47 ± 0.0024, respectively) (Fig. 2*E*), suggesting that this mutant motor is less efficient than WT MYO5B-MD. In contrast, MYO5B-MD(R824C) localized to the bases of the microvilli. The F-actin signal had a mean of 0.51 ± 0.0025 and MYO5B-MD(R824C) a mean of 0.26 ± 0.0096 (Fig. 2*F*). MYO5B-MD(R824C) signal did not extend as fully into microvilli as some of the other mutants, but was not primarily localized to the bases of microvilli, as seen with the rigor mutants. The localization of MYO5B-MD(R92C) was mainly at the tips of microvilli. The F-actin signal had a mean of 0.46 ± 0.0035 and MYO5B-MD(R92C) a mean of 0.54 ± 0.0085 (Fig. 2*J*). Liver mutants MYO5B-MD(C266R) and MYO5B-MD(R824C) have significantly decreased localization of highest intensity when compared with WT MYO5B-MD. Liver mutation MYO5B-MD(R92C) did not significantly change the localization of highest intensity when compared to the WT MYO5B-MD. Collectively, these results allow us to conclude that certain patient mutations behave in a similar manner to the characterized predicted MYO5B mutants.

### Use of fluorescence recovery after photobleaching to evaluate MVID-causing mutations on motor dynamics

**Figure 3:**
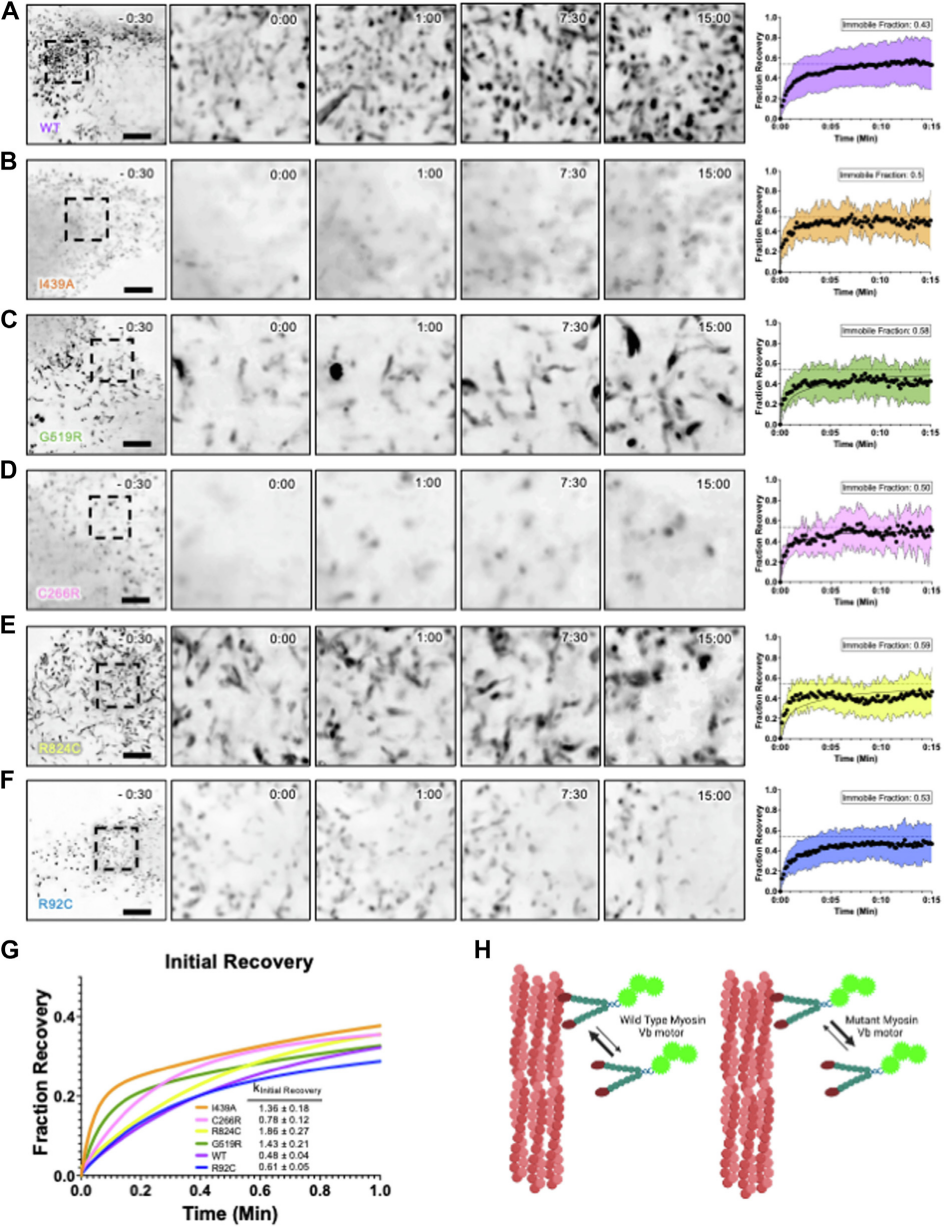
**Point mutations in the MYO5B motor with a predicted outcome disrupt normal MYO5B motor localization.***A,* FRAP of the WT MYO5B motor that localizes to the tips of microvilli. *B,* FRAP of the I439A MYO5B motor, the predicted uncoupled mutation. *C,* FRAP of the patient mutation, G519R. *D*, FRAP of the liver-specific patient mutation, C266R. *E,* FRAP of the liver-specific patient mutation, R824C. *F,* FRAP of the liver-specific patient mutation, R92C. *G,* this graph depicts an overlay of the initial recovery for all MYO5B motors assayed with FRAP. This graph shows that mutant motors I439A, G519R, and C266R have a steeper initial recovery than the WT motor. *H,* a cartoon depicting that the MYO5B mutations may increase the on/off rate of actin-binding kinetics than the WT (figure prepared with Biorender). Each condition has an N ≥ 11 cells from a replicate of three independent MatTek dishes. The 3x-Citrine-Myo5b motor overexpression is shown in *inverted grayscale*. The movies are deconvolved and have been histogram-corrected for bleaching. The scale bar represents 5 μm. FRAP, fluorescence recovery after photobleaching; MYOB, myosin 5b.

While images showing the localization of the truncated MYO5B motors along unipolar actin bundles give insights into whether the motor can translocate or not, they represent single snapshots in time. To gain insights into how patient mutations affect the dynamics of the MYO5B motor, we performed fluorescence recovery after photobleaching (FRAP) analysis on CL4 cells overexpressing MYO5B-MD constructs. For FRAP experiments, we focused specifically on patient mutants that were able to localize to microvilli. After photobleaching, WT MYO5B-MD demonstrated partial recovery (immobile fraction of 0.43 ± 0.16) (Fig. 3*A*). All the mutations tested resulted in an increase in the immobile fraction (Fig. 3, *A*–*F*), but interestingly resulted in an increase in the initial recovery (Fig. 3*G*). The uncoupled mutant, MYO5B-MD(I439A), was not able to recover to the same degree as WT MYO5B-MD and resulted in an increased immobile fraction of 0.50 ± 0.004 (Fig. 3*B*). The uncharacterized patient mutant MYO5B-MD(G519R) resulted in an immobile fraction of 0.58 ± 0.005, again not recovering as well as WT MYO5B-MD (Fig. 3*C*). Interestingly, FRAP of the liver-specific patient mutant MYO5B-MD(C266R) (0.50 ± 0.01), resulted in a immobile fraction similar to that of WT MYO5B-MD (Fig. 3*D*). The mutant MYO5B-MD(R824C) did not recover as well as WT MYO5B-MD and had an increased immobile fraction of 0.59 ± 0.004 (Fig. 3*E*). The mutant MYO5B-MD(R92C) resulted in an immobile fraction of 0.53 ± 0.004 (Fig. 3*F*).

In addition to the increase in the immobile fractions, we noted when comparing the initial recovery (up to the first minute), the mutant MYO5B constructs demonstrated faster kinetics than WT MYO5B-MD (Fig. 3*G*). To examine this further, we used the WT MYO5B-MD equation for the line of best fit from a nonlinear two-phase regression curve as the model and attempted to fit the data collected from the mutants to this model. Each mutant resulted in an R-squared value much smaller than WT MYO5B-MD: I439A: 0.086, G519R: 0.023, R824C: −0.03, and R92C: 0.1897 (Table 1). This further suggests that mutations in the motor domain impair the MYO5B motor dynamics and may increase the rate at which the mutant MYO5B constructs cycle on and off actin filaments.

**Table 1:** R^2^ value when fitted to the WT recovery curve model

	WT	I439A	G519R	C266R	R824C	R92C
R^2^ of WT curve fit	**0.20**	**0.09**	**0.02**	**−0.05**	**−0.03**	**−0.04**

The recovery curve for WT was used to create a model curve and then each of the mutants was fitted to the WT model. The R-squared values for fit of each mutant curve are reported.

## Discussion

MVID is a devastating disease for patients and has proven to be complex due to the variability between patients. We aimed to understand the impact of patient-derived MYO5B mutations on motor function, hopefully leading to insights about the disruption of normal cell biology. Due to the range of disease phenotypes observed in MVID patients, we developed an assay to characterize individual patient MYO5B motor mutants. Traditionally, myosin function and kinetics are studied using ATPase and *in vitro* motility assays, which require large quantities of highly purified protein. Our method enabled us to circumvent protein purification and single-molecule assays traditionally used to examine myosin motor function. With our assay, we were able to categorize the functional deficits in some of the patient mutants.

In our assay, we were able to provide further evidence that the P660L mutation is a rigor mutant, meaning it binds strongly to actin and rarely detaches. Additionally, we were able to categorize I408F as a potential actin nonbinder. Our assay also suggested that some of the liver specific mutations were partially functional, but were inefficient motors. While MYO5B function is important for intracellular trafficking in the epithelia of both the intestine and liver, MYO5B may have organ-specific functions that are impacted by one missense mutation but not another. It therefore remains unclear how alterations in the MYO5B protein can account for liver-specific phenotypes. Western blots indicated that some of the mutants were expressed at lower levels than our WT MYO5B-MD construct (Fig. S2). Notably, even in these cases, we were still able to identify individual cells with adequate fluorescence signal (a defined look-up table [LUT] peak between 150–250 intensity units) to allow for FRAP analysis. We cannot completely rule out that the change in expression levels in the mutants may have affected the FRAP data. We suspect that the endogenous full-length MYO5B within LLC-PK1-CL4 cells is not interfering with our overexpressed MYO5B-MD constructs since the MYO5B-MD construct can locate to tips of microvilli. The MYO5B-MD constructs can locate to the tips of microvilli because it does not have the tail domain to interact with Rab proteins like endogenous full-length MYO5B. Endogenous full-length MYO5B is located subapically below microvilli in tissue samples or with Rab11a-positive vesicles in cell lines. Although we do not expect it, we cannot fully rule out that endogenous MYO5B interacts with our truncated MYO5B-MD construct, which could affect the results of our data. The large scatter observed in the line scan data could be due to multiple factors, including mosaicism in the dimensions and orientation of microvilli on the apical surface of LLC-PK1-CL4 cells.

The FRAP experiments noted that disease-causing mutants in MYO5B motors lead to impaired motor dynamics. When examining the initial recovery (the first minute after bleaching) of the mutant MYO5B motors, they initially recovered faster than the WT MYO5B motor (Fig. 3*G*). Still, the end immobile fractions of the mutant motors were larger than WT (Fig. 3, *A*–*F*). This initial rapid recovery could suggest that these mutant motors do not bind as tightly to actin and cause more rapid actin kinetics (Fig. 3*H*). These more subtle changes noted by the FRAP experiments also suggest that although these mutations cause slight changes in motor dynamics, they lead to catastrophic disease. This phenomenon has been reported in other diseases caused by mutations in myosins. Mutations in the muscle myosin proteins are implicated in familial hypertrophic cardiomyopathy. One of the deadliest mutations in myosin heavy chain 7, R403Q, causes slight increases in the myosin kinetics, but these changes cause hypercontractility of the muscle, leading to the deadly disorder (68). A few of the mutants tested (I439A, G519R, R824C, and R92C) exhibited a faster initial recovery but resulted in a higher immobile fraction. The initial recovery and the immobile fraction occur on different time scales (seconds *versus* minutes). The initial recovery could reflect the kinetics of motor domain binding to and detachment from actin, which is expected to occur on a similar time scale. In contrast, the immobile fraction is measured after 15 min of recovery and is likely controlled by a distinct process. Previous studies leveraging MYO5B KO models helped establish the foundational knowledge of MVID disease pathology. A newer mouse model mimicking a patient with a missense mutation, the G519R mouse model (50), has established that retention of an impaired MYO5B may result in more challenges to overcome disease pathology than complete loss of MYO5B. We established a method to test the motor function of MYO5B independent of cargo binding to investigate the effect of patient mutations on motor function specifically. Our studies show that patient missense mutations exhibit a range of impacts on motor function and subtle changes to motor dynamics.

## Experimental procedures

### Cell culture

LLC-PK1-CL4 cells (male porcine proximal kidney tubule cells, ATCC CRL-1849) were cultured in 1X high glucose Dulbecco's modified Eagle's medium containing 2 mM L-glutamine (Corning #10-013-CV) with high glucose, supplemented with 10% fetal bovine serum (Gibco, #10438–026) and 1% L-glutamine (Corning # 25-005-CI). Cells were tested for *mycoplasma* using the MycoAlert PLUS *Mycoplasma* Detection Kit (Lonza #LT07-710).

### Cloning and constructs

The triple tandem mCitrine C terminus tagged MYO5B motor (1-1016X), referred to as MYO5B-MD, and was previously described, as well as the P660L mutant (47). A plasmid containing the 3xCitrine tag was originally obtained as a gift from the Verhey lab (69). The 3xCitrine tag was cloned into a mammalian expression vector with a pCMV promotor. The human sequence for MYO5B, which includes the motor head, neck domain, and part of the coiled-coil domain (a.a. 1-1015), was then inserted, creating a C-terminal in-frame fusion to the 3x-citrine tag. Mutant constructs were made using a single mutagenesis primer and the QuickChange Lightning Multi Site-directed Mutagenesis kit from Agilent (Cat #210515-5). Colonies were selected using the bacterial resistance kanamycin, and sequencing was used to confirm all constructs. Site-directed mutagenesis was then performed on the WT MYO5B motor-3xCitrine plasmid to create plasmids for each of the predicted mutations and MVID-causing MYO5B mutations; N208A, E443A, I439A, G519R, R824C, C266R, I408F, and R92C using Quickchange Multi systems (Agilent). Mutagenesis primers are included in Table S3. All the MYO5B motor-3xCitrine constructs have G418/neomycin selection as part of the backbone. The models of MYO5B shown in Figures 1*A* and 2*A* were created using the sequence of human MYO5B from UniProt (Q9ULV0) and AlphaFold Structure (AF-Q9ULV0-v4).

### Transient transfections and cell fixation

LLC-PK1-CL4 cells were seeded onto plasma cleaned 35 mm glass-bottom dishes (CellVis, D35-20-1.5-N) and transfected at around 60 to 70% confluency using Lipofectamine2000 according to manufacturer instructions with 1X OptiMEM (Gibco, #31985-070). Media was changed 4 h after transfection. Cells were used for live-cell or fixed imaging or Western blots for the highest expression level between 24 and 48 h posttransfection. For fixed images, CL4 cells plated on MatTek dishes 24 to 48 h posttransfection were first rinsed in warm 1X PBS and fixed using 4% paraformaldehyde for 20 min. Following fixations, CL4 cells were washed with PBS three times for 5 min each. The fixed CL4 cells were then permeabilized with 0.1% Triton X-100 for 10 min at room temperature. 405 phalloidin was then diluted into PBS with 1% bovine serum albumin and incubated on cells for 1 h at room temperature. The cells were washed with PBS thrice and left in PBS for fixed imaging.

### Microscopy

For fixed cell imaging, a spinning disc confocal microscope was used; Nikon Ti2 inverted light microscope equipped with a Yokogawa CSU-W1/SORA spinning disk head, an ORCA-fusionBT (C15440) camera, 405 nm, 488 nm, 561 nm, and 647 nm excitation lasers, and a 100x/1.45 NA Plan Apo λD objective. When imaging, cells with moderate expression were chosen to peak within a LUTs range of 150 to –250 and all images were taken at the same laser intensity and exposure. A Z-stack with a step size of 0.03 μm was used to image from the top of the cell to the bottom.

For live imaging, spinning disc confocal microscopy was done using a Nikon Ti2 inverted light microscope equipped with a Yokogawa CSU-W1/SORA spinning disk head, an ORCA-fusionBT (C15440) camera, 405 nm, 488 nm, 561 nm, and 647 nm excitation lasers, and a 100x/1.45 NA Plan Apo λD objective. Before imaging, cell media was aspirated off, and the cells were washed with warmed 1xPBS. 2.5 ml of FluoroBrite imaging media (Gibco #A18967-01) supplemented with 10% fetal bovine serum was added to the glass-bottom MatTek dishes for live imaging. Cells were maintained in a stage top incubator at 37 °C for live imaging with 5% CO2 (Tokai Hit). The 405 nm stimulation laser was used for FRAP experiments with a 100x/1.49 NA total internal reflection flourescence objective. When imaging, cells with moderate expression were chosen using a LUT range of 150 to 250 and all images were taken using the same laser and camera settings. A square region of interest (ROI) was drawn in Nikon Elements to capture FRAP. The cells were first imaged at 10-s intervals for four frames prebleach within a 5 μm z-stack. The bleach ROI was stimulated with a 405 laser at 2% with a dwell of 50 μs. Fluorescence recovery was then monitored by images taken in 10-s intervals for 15 min. Movies were compared across conditions and were collected using matching laser power and exposure times.

### Quantification and statistical analysis

Localization of MYO5B-MD-CC-3x-Citrine along microvilli: Crops with a short width (5–10 μm) were taken from the XY view of images. The crops were then viewed in the orthogonal view (XZ or YZ). The orthogonal view was then condensed into a maximum intensity projection view and saved. This allowed for the whole microvilli to be viewed. In FIJI (https://imagej.net/software/fiji), a line scan with a width of two was drawn from the base of visible microvilli (that had no other overlapped microvilli) using the phalloidin channel. The signal distribution along the length was measured in the phalloidin channel and then the MYO5B-MD-CC-3x-Citrine channel. The length and signal intensities (for both phalloidin and MYO5B motor) were normalized and then graphed with the length of microvilli as the *x*-axis, the base of the microvilli being at the 0 point. The shape and peak of the MYO5B-MD-CC-3x-Citrine best fit can then be compared to the shape and peak of the phalloidin best fit. These measurements were then normalized for both length and fluorescence intensity and plotted. Prism was then used to determine if a Gaussian or straight line better fit the data. Additionally, the normalized position of the highest intensity was gathered from each microvilli measurement and then averaged for each cell measured. This position was then graphed in order to compare different MYO5B-MD-CC-3x-Citrine constructs using a one-way ANOVA statistical test.

#### FRAP

A background ROI and reference ROI were used for photobleaching and background fluorescence. Fraction recovery over time was calculated from (bleach ROI—background)/(reference ROI—background). Recovery curves were fitted with a single-phase association equation in prism, and the immobile fraction was calculated from 1 minus the plateau. Images shown in Figure 3 were deconvolved in Nikon Elements for presentation clarity; however, all analyzed measurements presented in the FRAP plots were taken from raw, unprocessed movies. Additionally, prism was used to create a 3xCitrine WT MYO5B motor curve standard; the mutant data was then attempted to be fitted to the WT standard, and the R values were reported.

#### Western blot

For LLC-PK1-CL4 cell Western blots, the cells were transiently transfected as described above the day before collection in a 6-well plate. Cell at 75 to 90% confluency were first washed with PBS then lysed in 100 ul of hot lysis buffer (60 mM Tris pH6.8, 10 mM EDTA, 2% SDS) and scraped off the plate. Samples were heated at 65 °C for 10 min and then sonicated for 20 s. Samples were spun down at 13 k rpm for 10 min at 4 °C. The protein concentration was then quantified using a bicinchonic acid assay, and samples were diluted to match the lowest sample protein concentration with tris-buffered saline. Lysates were run on 4 to 12% polyacrylamide gels and then transferred onto Odyssey nitrocellulose membrane (LiCor) for 1 h at 75 V. Membranes were blocked with milk tris-buffered saline buffer and probed with rabbit anti-MYO5B (Novus AB_11034537) and mouse anti-β Actin (Sigma AB_476743). Detection was done with Odyssey anti-rabbit 800 nm secondary antibody (LiCor) and Odyssey anti-mouse 680 nm secondary antibody (LiCor). Blots were imaged using a LiCor Odyssey Fc.

## Data availability

All data in this investigation are contained in this article.

## Supporting information

This article contains supporting information.

## Conflict of interest

The authors declare that they have no conflicts of interest with the contents of this article.
