# Supplementary material for: Microvillus inclusion disease-causing MYO5B point mutations exert differential effects on motor function
Source: J Biol Chem. 2025 Feb 18;301(4):108328. doi: 10.1016/j.jbc.2025.108328 (PMC11964754; doi:10.1016/j.jbc.2025.108328)
Supplement: Supplemental Figures and Legends [file mmc1.pptx]

## Slide 1
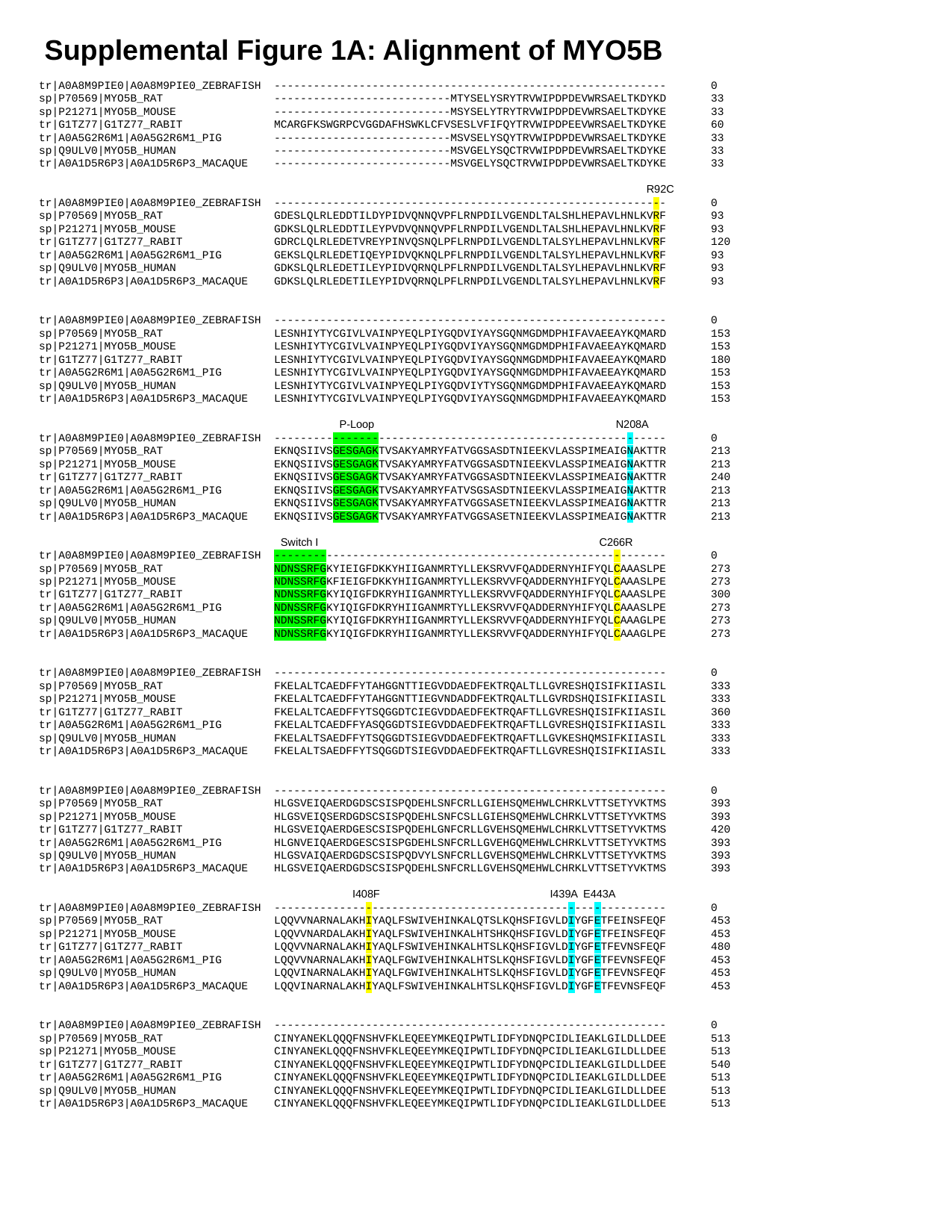

Supplemental Figure 1A: Alignment of MYO5B
tr|A0A8M9PIE0|A0A8M9PIE0_ZEBRAFISH ------------------------------------------------------------	0
sp|P70569|MYO5B_RAT ---------------------------MTYSELYSRYTRVWIPDPDEVWRSAELTKDYKD	33
sp|P21271|MYO5B_MOUSE ---------------------------MSYSELYTRYTRVWIPDPDEVWRSAELTKDYKE	33
tr|G1TZ77|G1TZ77_RABIT MCARGFKSWGRPCVGGDAFHSWKLCFVSESLVFIFQYTRVWIPDPEEVWRSAELTKDYKE	60
tr|A0A5G2R6M1|A0A5G2R6M1_PIG ---------------------------MSVSELYSQYTRVWIPDPDEVWRSAELTKDYKE	33
sp|Q9ULV0|MYO5B_HUMAN ---------------------------MSVGELYSQCTRVWIPDPDEVWRSAELTKDYKE	33
tr|A0A1D5R6P3|A0A1D5R6P3_MACAQUE ---------------------------MSVGELYSQCTRVWIPDPDEVWRSAELTKDYKE	33
 R92C
tr|A0A8M9PIE0|A0A8M9PIE0_ZEBRAFISH ------------------------------------------------------------	0
sp|P70569|MYO5B_RAT GDESLQLRLEDDTILDYPIDVQNNQVPFLRNPDILVGENDLTALSHLHEPAVLHNLKVRF	93
sp|P21271|MYO5B_MOUSE GDKSLQLRLEDDTILEYPVDVQNNQVPFLRNPDILVGENDLTALSHLHEPAVLHNLKVRF	93
tr|G1TZ77|G1TZ77_RABIT GDRCLQLRLEDETVREYPINVQSNQLPFLRNPDILVGENDLTALSYLHEPAVLHNLKVRF	120
tr|A0A5G2R6M1|A0A5G2R6M1_PIG GEKSLQLRLEDETIQEYPIDVQKNQLPFLRNPDILVGENDLTALSYLHEPAVLHNLKVRF	93
sp|Q9ULV0|MYO5B_HUMAN GDKSLQLRLEDETILEYPIDVQRNQLPFLRNPDILVGENDLTALSYLHEPAVLHNLKVRF	93
tr|A0A1D5R6P3|A0A1D5R6P3_MACAQUE GDKSLQLRLEDETILEYPIDVQRNQLPFLRNPDILVGENDLTALSYLHEPAVLHNLKVRF	93
tr|A0A8M9PIE0|A0A8M9PIE0_ZEBRAFISH ------------------------------------------------------------	0
sp|P70569|MYO5B_RAT LESNHIYTYCGIVLVAINPYEQLPIYGQDVIYAYSGQNMGDMDPHIFAVAEEAYKQMARD	153
sp|P21271|MYO5B_MOUSE LESNHIYTYCGIVLVAINPYEQLPIYGQDVIYAYSGQNMGDMDPHIFAVAEEAYKQMARD	153
tr|G1TZ77|G1TZ77_RABIT LESNHIYTYCGIVLVAINPYEQLPIYGQDVIYAYSGQNMGDMDPHIFAVAEEAYKQMARD	180
tr|A0A5G2R6M1|A0A5G2R6M1_PIG LESNHIYTYCGIVLVAINPYEQLPIYGQDVIYAYSGQNMGDMDPHIFAVAEEAYKQMARD	153
sp|Q9ULV0|MYO5B_HUMAN LESNHIYTYCGIVLVAINPYEQLPIYGQDVIYTYSGQNMGDMDPHIFAVAEEAYKQMARD	153
tr|A0A1D5R6P3|A0A1D5R6P3_MACAQUE LESNHIYTYCGIVLVAINPYEQLPIYGQDVIYAYSGQNMGDMDPHIFAVAEEAYKQMARD	153
 P-Loop N208A
tr|A0A8M9PIE0|A0A8M9PIE0_ZEBRAFISH ------------------------------------------------------------	0
sp|P70569|MYO5B_RAT EKNQSIIVSGESGAGKTVSAKYAMRYFATVGGSASDTNIEEKVLASSPIMEAIGNAKTTR	213
sp|P21271|MYO5B_MOUSE EKNQSIIVSGESGAGKTVSAKYAMRYFATVGGSASDTNIEEKVLASSPIMEAIGNAKTTR	213
tr|G1TZ77|G1TZ77_RABIT EKNQSIIVSGESGAGKTVSAKYAMRYFATVGGSASDTNIEEKVLASSPIMEAIGNAKTTR	240
tr|A0A5G2R6M1|A0A5G2R6M1_PIG EKNQSIIVSGESGAGKTVSAKYAMRYFATVSGSASDTNIEEKVLASSPIMEAIGNAKTTR	213
sp|Q9ULV0|MYO5B_HUMAN EKNQSIIVSGESGAGKTVSAKYAMRYFATVGGSASETNIEEKVLASSPIMEAIGNAKTTR	213
tr|A0A1D5R6P3|A0A1D5R6P3_MACAQUE EKNQSIIVSGESGAGKTVSAKYAMRYFATVGGSASETNIEEKVLASSPIMEAIGNAKTTR	213
 Switch I C266R
tr|A0A8M9PIE0|A0A8M9PIE0_ZEBRAFISH ------------------------------------------------------------	0
sp|P70569|MYO5B_RAT NDNSSRFGKYIEIGFDKKYHIIGANMRTYLLEKSRVVFQADDERNYHIFYQLCAAASLPE	273
sp|P21271|MYO5B_MOUSE NDNSSRFGKFIEIGFDKKYHIIGANMRTYLLEKSRVVFQADDERNYHIFYQLCAAASLPE	273
tr|G1TZ77|G1TZ77_RABIT NDNSSRFGKYIQIGFDKRYHIIGANMRTYLLEKSRVVFQADDERNYHIFYQLCAAASLPE	300
tr|A0A5G2R6M1|A0A5G2R6M1_PIG NDNSSRFGKYIQIGFDKRYHIIGANMRTYLLEKSRVVFQADDERNYHIFYQLCAAASLPE	273
sp|Q9ULV0|MYO5B_HUMAN NDNSSRFGKYIQIGFDKRYHIIGANMRTYLLEKSRVVFQADDERNYHIFYQLCAAAGLPE	273
tr|A0A1D5R6P3|A0A1D5R6P3_MACAQUE NDNSSRFGKYIQIGFDKRYHIIGANMRTYLLEKSRVVFQADDERNYHIFYQLCAAAGLPE	273
tr|A0A8M9PIE0|A0A8M9PIE0_ZEBRAFISH ------------------------------------------------------------	0
sp|P70569|MYO5B_RAT FKELALTCAEDFFYTAHGGNTTIEGVDDAEDFEKTRQALTLLGVRESHQISIFKIIASIL	333
sp|P21271|MYO5B_MOUSE FKELALTCAEDFFYTAHGGNTTIEGVNDADDFEKTRQALTLLGVRDSHQISIFKIIASIL	333
tr|G1TZ77|G1TZ77_RABIT FKELALTCAEDFFYTSQGGDTCIEGVDDAEDFEKTRQAFTLLGVRESHQISIFKIIASIL	360
tr|A0A5G2R6M1|A0A5G2R6M1_PIG FKELALTCAEDFFYASQGGDTSIEGVDDAEDFEKTRQAFTLLGVRESHQISIFKIIASIL	333
sp|Q9ULV0|MYO5B_HUMAN FKELALTSAEDFFYTSQGGDTSIEGVDDAEDFEKTRQAFTLLGVKESHQMSIFKIIASIL	333
tr|A0A1D5R6P3|A0A1D5R6P3_MACAQUE FKELALTSAEDFFYTSQGGDTSIEGVDDAEDFEKTRQAFTLLGVRESHQISIFKIIASIL	333
tr|A0A8M9PIE0|A0A8M9PIE0_ZEBRAFISH ------------------------------------------------------------	0
sp|P70569|MYO5B_RAT HLGSVEIQAERDGDSCSISPQDEHLSNFCRLLGIEHSQMEHWLCHRKLVTTSETYVKTMS	393
sp|P21271|MYO5B_MOUSE HLGSVEIQSERDGDSCSISPQDEHLSNFCSLLGIEHSQMEHWLCHRKLVTTSETYVKTMS	393
tr|G1TZ77|G1TZ77_RABIT HLGSVEIQAERDGESCSISPQDEHLGNFCRLLGVEHSQMEHWLCHRKLVTTSETYVKTMS	420
tr|A0A5G2R6M1|A0A5G2R6M1_PIG HLGNVEIQAERDGESCSISPGDEHLSNFCRLLGVEHGQMEHWLCHRKLVTTSETYVKTMS	393
sp|Q9ULV0|MYO5B_HUMAN HLGSVAIQAERDGDSCSISPQDVYLSNFCRLLGVEHSQMEHWLCHRKLVTTSETYVKTMS	393
tr|A0A1D5R6P3|A0A1D5R6P3_MACAQUE HLGSVEIQAERDGDSCSISPQDEHLSNFCRLLGVEHSQMEHWLCHRKLVTTSETYVKTMS	393
 I408F I439A E443A
tr|A0A8M9PIE0|A0A8M9PIE0_ZEBRAFISH ------------------------------------------------------------	0
sp|P70569|MYO5B_RAT LQQVVNARNALAKHIYAQLFSWIVEHINKALQTSLKQHSFIGVLDIYGFETFEINSFEQF	453
sp|P21271|MYO5B_MOUSE LQQVVNARDALAKHIYAQLFSWIVEHINKALHTSHKQHSFIGVLDIYGFETFEINSFEQF	453
tr|G1TZ77|G1TZ77_RABIT LQQVVNARNALAKHIYAQLFSWIVEHINKALHTSLKQHSFIGVLDIYGFETFEVNSFEQF	480
tr|A0A5G2R6M1|A0A5G2R6M1_PIG LQQVVNARNALAKHIYAQLFGWIVEHINKALHTSLKQHSFIGVLDIYGFETFEVNSFEQF	453
sp|Q9ULV0|MYO5B_HUMAN LQQVINARNALAKHIYAQLFGWIVEHINKALHTSLKQHSFIGVLDIYGFETFEVNSFEQF	453
tr|A0A1D5R6P3|A0A1D5R6P3_MACAQUE LQQVINARNALAKHIYAQLFSWIVEHINKALHTSLKQHSFIGVLDIYGFETFEVNSFEQF	453
tr|A0A8M9PIE0|A0A8M9PIE0_ZEBRAFISH ------------------------------------------------------------	0
sp|P70569|MYO5B_RAT CINYANEKLQQQFNSHVFKLEQEEYMKEQIPWTLIDFYDNQPCIDLIEAKLGILDLLDEE	513
sp|P21271|MYO5B_MOUSE CINYANEKLQQQFNSHVFKLEQEEYMKEQIPWTLIDFYDNQPCIDLIEAKLGILDLLDEE	513
tr|G1TZ77|G1TZ77_RABIT CINYANEKLQQQFNSHVFKLEQEEYMKEQIPWTLIDFYDNQPCIDLIEAKLGILDLLDEE	540
tr|A0A5G2R6M1|A0A5G2R6M1_PIG CINYANEKLQQQFNSHVFKLEQEEYMKEQIPWTLIDFYDNQPCIDLIEAKLGILDLLDEE	513
sp|Q9ULV0|MYO5B_HUMAN CINYANEKLQQQFNSHVFKLEQEEYMKEQIPWTLIDFYDNQPCIDLIEAKLGILDLLDEE	513
tr|A0A1D5R6P3|A0A1D5R6P3_MACAQUE CINYANEKLQQQFNSHVFKLEQEEYMKEQIPWTLIDFYDNQPCIDLIEAKLGILDLLDEE	513

## Slide 2
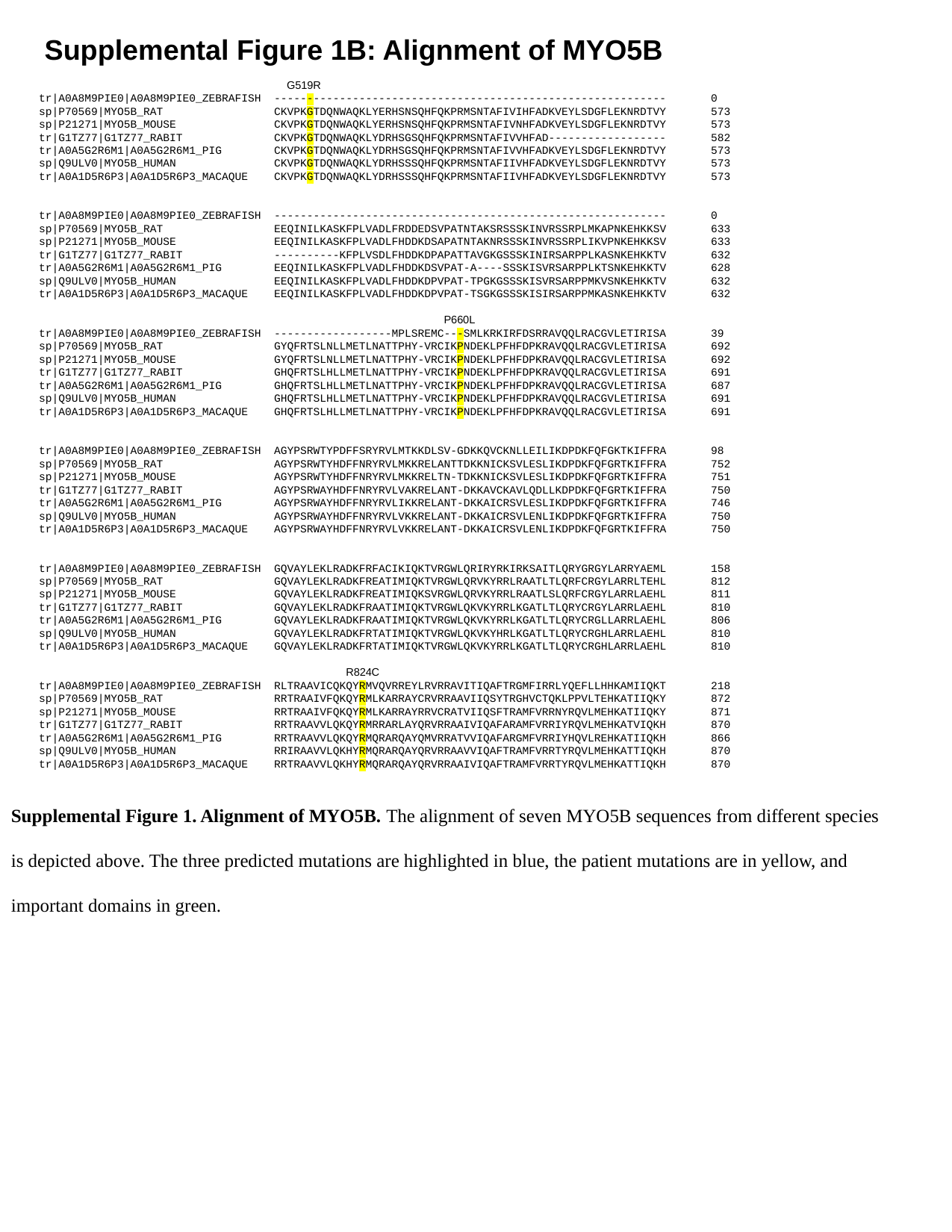

Supplemental Figure 1B: Alignment of MYO5B
 G519R
tr|A0A8M9PIE0|A0A8M9PIE0_ZEBRAFISH ------------------------------------------------------------	0
sp|P70569|MYO5B_RAT CKVPKGTDQNWAQKLYERHSNSQHFQKPRMSNTAFIVIHFADKVEYLSDGFLEKNRDTVY	573
sp|P21271|MYO5B_MOUSE CKVPKGTDQNWAQKLYERHSNSQHFQKPRMSNTAFIVNHFADKVEYLSDGFLEKNRDTVY	573
tr|G1TZ77|G1TZ77_RABIT CKVPKGTDQNWAQKLYDRHSGSQHFQKPRMSNTAFIVVHFAD------------------	582
tr|A0A5G2R6M1|A0A5G2R6M1_PIG CKVPKGTDQNWAQKLYDRHSGSQHFQKPRMSNTAFIVVHFADKVEYLSDGFLEKNRDTVY	573
sp|Q9ULV0|MYO5B_HUMAN CKVPKGTDQNWAQKLYDRHSSSQHFQKPRMSNTAFIIVHFADKVEYLSDGFLEKNRDTVY	573
tr|A0A1D5R6P3|A0A1D5R6P3_MACAQUE CKVPKGTDQNWAQKLYDRHSSSQHFQKPRMSNTAFIIVHFADKVEYLSDGFLEKNRDTVY	573
tr|A0A8M9PIE0|A0A8M9PIE0_ZEBRAFISH ------------------------------------------------------------	0
sp|P70569|MYO5B_RAT EEQINILKASKFPLVADLFRDDEDSVPATNTAKSRSSSKINVRSSRPLMKAPNKEHKKSV	633
sp|P21271|MYO5B_MOUSE EEQINILKASKFPLVADLFHDDKDSAPATNTAKNRSSSKINVRSSRPLIKVPNKEHKKSV	633
tr|G1TZ77|G1TZ77_RABIT ----------KFPLVSDLFHDDKDPAPATTAVGKGSSSKINIRSARPPLKASNKEHKKTV	632
tr|A0A5G2R6M1|A0A5G2R6M1_PIG EEQINILKASKFPLVADLFHDDKDSVPAT-A----SSSKISVRSARPPLKTSNKEHKKTV	628
sp|Q9ULV0|MYO5B_HUMAN EEQINILKASKFPLVADLFHDDKDPVPAT-TPGKGSSSKISVRSARPPMKVSNKEHKKTV	632
tr|A0A1D5R6P3|A0A1D5R6P3_MACAQUE EEQINILKASKFPLVADLFHDDKDPVPAT-TSGKGSSSKISIRSARPPMKASNKEHKKTV	632
 P660L
tr|A0A8M9PIE0|A0A8M9PIE0_ZEBRAFISH ------------------MPLSREMC---SMLKRKIRFDSRRAVQQLRACGVLETIRISA	39
sp|P70569|MYO5B_RAT GYQFRTSLNLLMETLNATTPHY-VRCIKPNDEKLPFHFDPKRAVQQLRACGVLETIRISA	692
sp|P21271|MYO5B_MOUSE GYQFRTSLNLLMETLNATTPHY-VRCIKPNDEKLPFHFDPKRAVQQLRACGVLETIRISA	692
tr|G1TZ77|G1TZ77_RABIT GHQFRTSLHLLMETLNATTPHY-VRCIKPNDEKLPFHFDPKRAVQQLRACGVLETIRISA	691
tr|A0A5G2R6M1|A0A5G2R6M1_PIG GHQFRTSLHLLMETLNATTPHY-VRCIKPNDEKLPFHFDPKRAVQQLRACGVLETIRISA	687
sp|Q9ULV0|MYO5B_HUMAN GHQFRTSLHLLMETLNATTPHY-VRCIKPNDEKLPFHFDPKRAVQQLRACGVLETIRISA	691
tr|A0A1D5R6P3|A0A1D5R6P3_MACAQUE GHQFRTSLHLLMETLNATTPHY-VRCIKPNDEKLPFHFDPKRAVQQLRACGVLETIRISA	691
tr|A0A8M9PIE0|A0A8M9PIE0_ZEBRAFISH AGYPSRWTYPDFFSRYRVLMTKKDLSV-GDKKQVCKNLLEILIKDPDKFQFGKTKIFFRA	98
sp|P70569|MYO5B_RAT AGYPSRWTYHDFFNRYRVLMKKRELANTTDKKNICKSVLESLIKDPDKFQFGRTKIFFRA	752
sp|P21271|MYO5B_MOUSE AGYPSRWTYHDFFNRYRVLMKKRELTN-TDKKNICKSVLESLIKDPDKFQFGRTKIFFRA	751
tr|G1TZ77|G1TZ77_RABIT AGYPSRWAYHDFFNRYRVLVAKRELANT-DKKAVCKAVLQDLLKDPDKFQFGRTKIFFRA	750
tr|A0A5G2R6M1|A0A5G2R6M1_PIG AGYPSRWAYHDFFNRYRVLIKKRELANT-DKKAICRSVLESLIKDPDKFQFGRTKIFFRA	746
sp|Q9ULV0|MYO5B_HUMAN AGYPSRWAYHDFFNRYRVLVKKRELANT-DKKAICRSVLENLIKDPDKFQFGRTKIFFRA	750
tr|A0A1D5R6P3|A0A1D5R6P3_MACAQUE AGYPSRWAYHDFFNRYRVLVKKRELANT-DKKAICRSVLENLIKDPDKFQFGRTKIFFRA	750
tr|A0A8M9PIE0|A0A8M9PIE0_ZEBRAFISH GQVAYLEKLRADKFRFACIKIQKTVRGWLQRIRYRKIRKSAITLQRYGRGYLARRYAEML	158
sp|P70569|MYO5B_RAT GQVAYLEKLRADKFREATIMIQKTVRGWLQRVKYRRLRAATLTLQRFCRGYLARRLTEHL	812
sp|P21271|MYO5B_MOUSE GQVAYLEKLRADKFREATIMIQKSVRGWLQRVKYRRLRAATLSLQRFCRGYLARRLAEHL	811
tr|G1TZ77|G1TZ77_RABIT GQVAYLEKLRADKFRAATIMIQKTVRGWLQKVKYRRLKGATLTLQRYCRGYLARRLAEHL	810
tr|A0A5G2R6M1|A0A5G2R6M1_PIG GQVAYLEKLRADKFRAATIMIQKTVRGWLQKVKYRRLKGATLTLQRYCRGLLARRLAEHL	806
sp|Q9ULV0|MYO5B_HUMAN GQVAYLEKLRADKFRTATIMIQKTVRGWLQKVKYHRLKGATLTLQRYCRGHLARRLAEHL	810
tr|A0A1D5R6P3|A0A1D5R6P3_MACAQUE GQVAYLEKLRADKFRTATIMIQKTVRGWLQKVKYRRLKGATLTLQRYCRGHLARRLAEHL	810
 R824C
tr|A0A8M9PIE0|A0A8M9PIE0_ZEBRAFISH RLTRAAVICQKQYRMVQVRREYLRVRRAVITIQAFTRGMFIRRLYQEFLLHHKAMIIQKT	218
sp|P70569|MYO5B_RAT RRTRAAIVFQKQYRMLKARRAYCRVRRAAVIIQSYTRGHVCTQKLPPVLTEHKATIIQKY	872
sp|P21271|MYO5B_MOUSE RRTRAAIVFQKQYRMLKARRAYRRVCRATVIIQSFTRAMFVRRNYRQVLMEHKATIIQKY	871
tr|G1TZ77|G1TZ77_RABIT RRTRAAVVLQKQYRMRRARLAYQRVRRAAIVIQAFARAMFVRRIYRQVLMEHKATVIQKH	870
tr|A0A5G2R6M1|A0A5G2R6M1_PIG RRTRAAVVLQKQYRMQRARQAYQMVRRATVVIQAFARGMFVRRIYHQVLREHKATIIQKH	866
sp|Q9ULV0|MYO5B_HUMAN RRIRAAVVLQKHYRMQRARQAYQRVRRAAVVIQAFTRAMFVRRTYRQVLMEHKATTIQKH	870
tr|A0A1D5R6P3|A0A1D5R6P3_MACAQUE RRTRAAVVLQKHYRMQRARQAYQRVRRAAIVIQAFTRAMFVRRTYRQVLMEHKATTIQKH	870
Supplemental Figure 1. Alignment of MYO5B. The alignment of seven MYO5B sequences from different species is depicted above. The three predicted mutations are highlighted in blue, the patient mutations are in yellow, and important domains in green.

## Slide 3
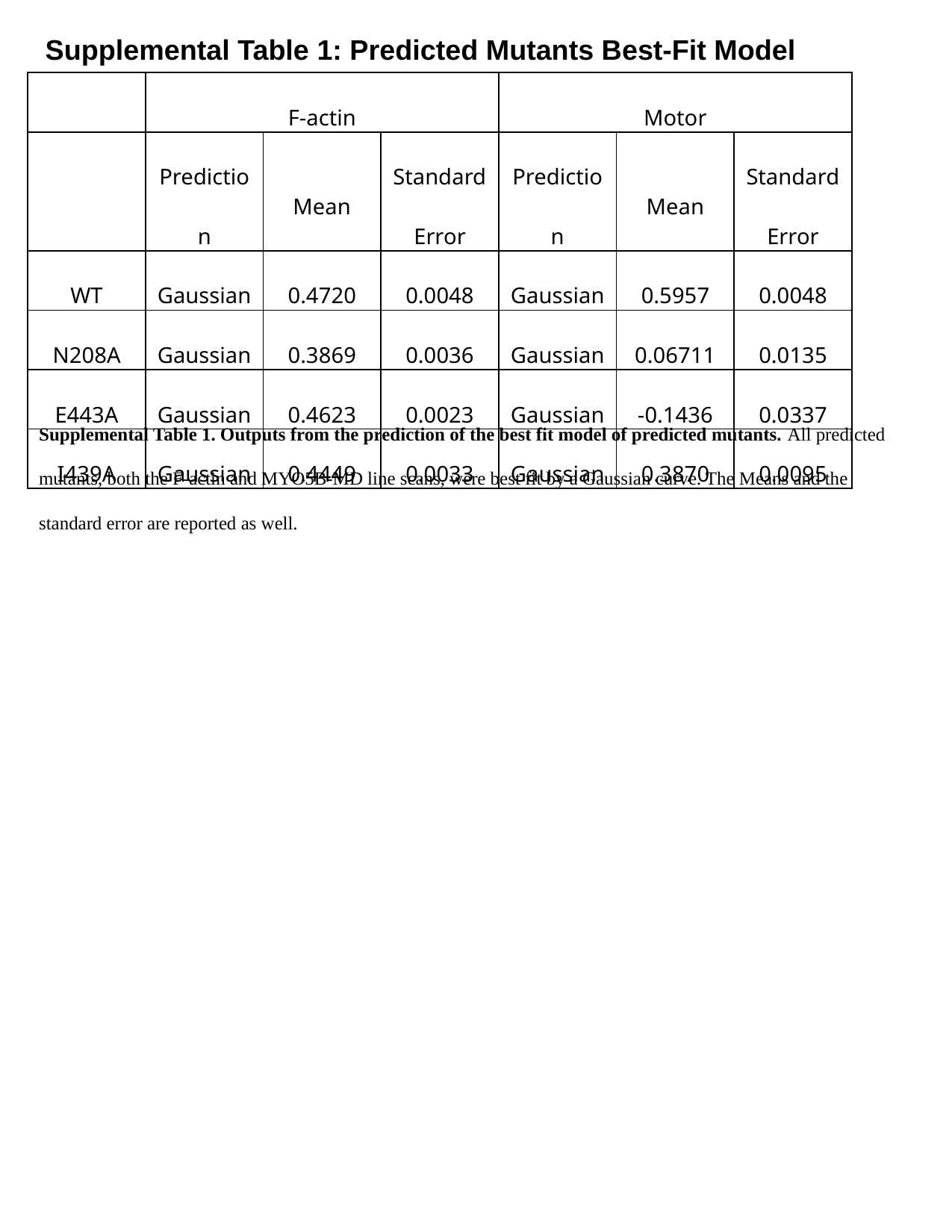

Supplemental Table 1: Predicted Mutants Best-Fit Model
| | F-actin | | | Motor | | |
| --- | --- | --- | --- | --- | --- | --- |
| | Prediction | Mean | Standard Error | Prediction | Mean | Standard Error |
| WT | Gaussian | 0.4720 | 0.0048 | Gaussian | 0.5957 | 0.0048 |
| N208A | Gaussian | 0.3869 | 0.0036 | Gaussian | 0.06711 | 0.0135 |
| E443A | Gaussian | 0.4623 | 0.0023 | Gaussian | -0.1436 | 0.0337 |
| I439A | Gaussian | 0.4449 | 0.0033 | Gaussian | 0.3870 | 0.0095 |
Supplemental Table 1. Outputs from the prediction of the best fit model of predicted mutants. All predicted mutants, both the F-actin and MYO5B-MD line scans, were best fit by a Gaussian curve. The Means and the standard error are reported as well.

## Slide 4
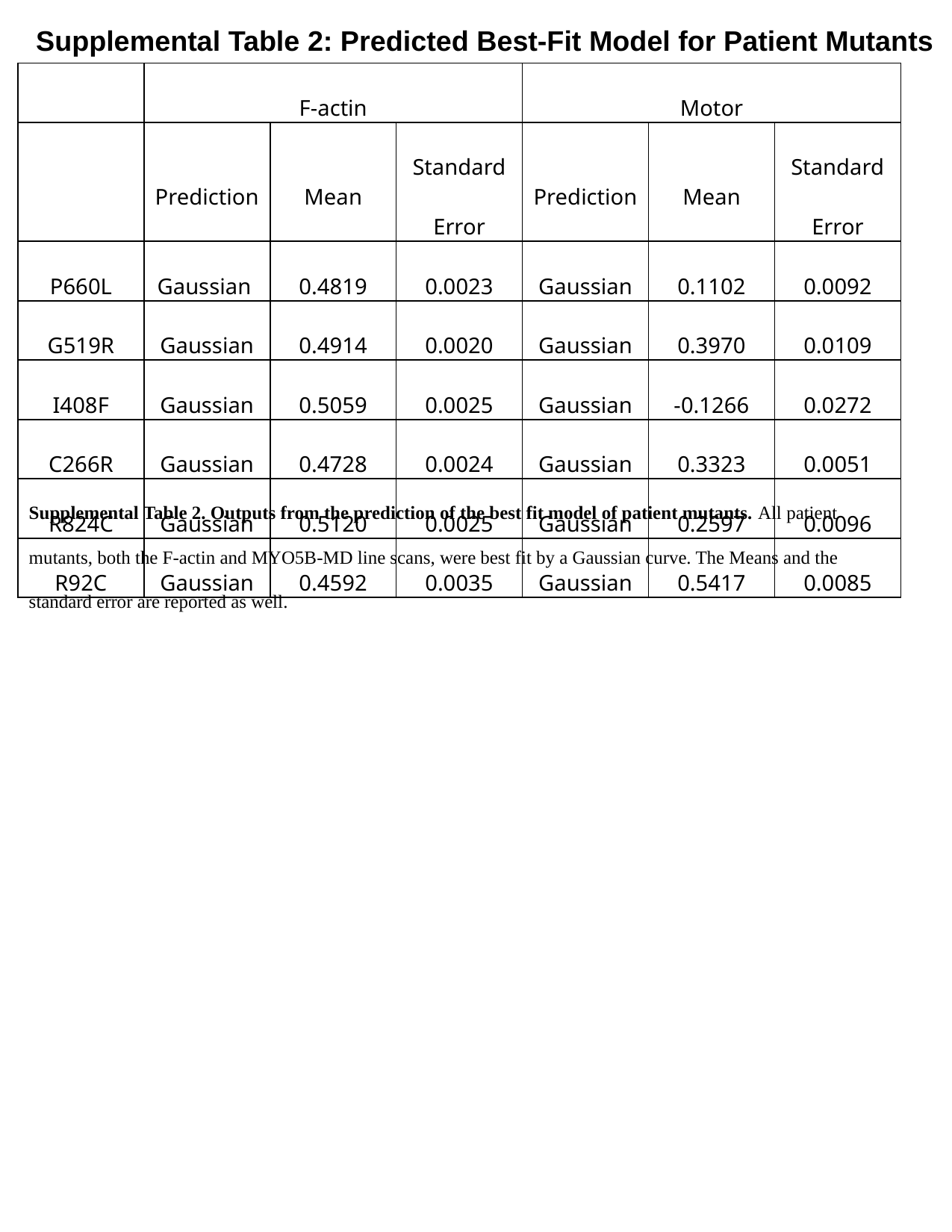

Supplemental Table 2: Predicted Best-Fit Model for Patient Mutants
| | F-actin | | | Motor | | |
| --- | --- | --- | --- | --- | --- | --- |
| | Prediction | Mean | Standard Error | Prediction | Mean | Standard Error |
| P660L | Gaussian | 0.4819 | 0.0023 | Gaussian | 0.1102 | 0.0092 |
| G519R | Gaussian | 0.4914 | 0.0020 | Gaussian | 0.3970 | 0.0109 |
| I408F | Gaussian | 0.5059 | 0.0025 | Gaussian | -0.1266 | 0.0272 |
| C266R | Gaussian | 0.4728 | 0.0024 | Gaussian | 0.3323 | 0.0051 |
| R824C | Gaussian | 0.5120 | 0.0025 | Gaussian | 0.2597 | 0.0096 |
| R92C | Gaussian | 0.4592 | 0.0035 | Gaussian | 0.5417 | 0.0085 |
Supplemental Table 2. Outputs from the prediction of the best fit model of patient mutants. All patient mutants, both the F-actin and MYO5B-MD line scans, were best fit by a Gaussian curve. The Means and the standard error are reported as well.

## Slide 5
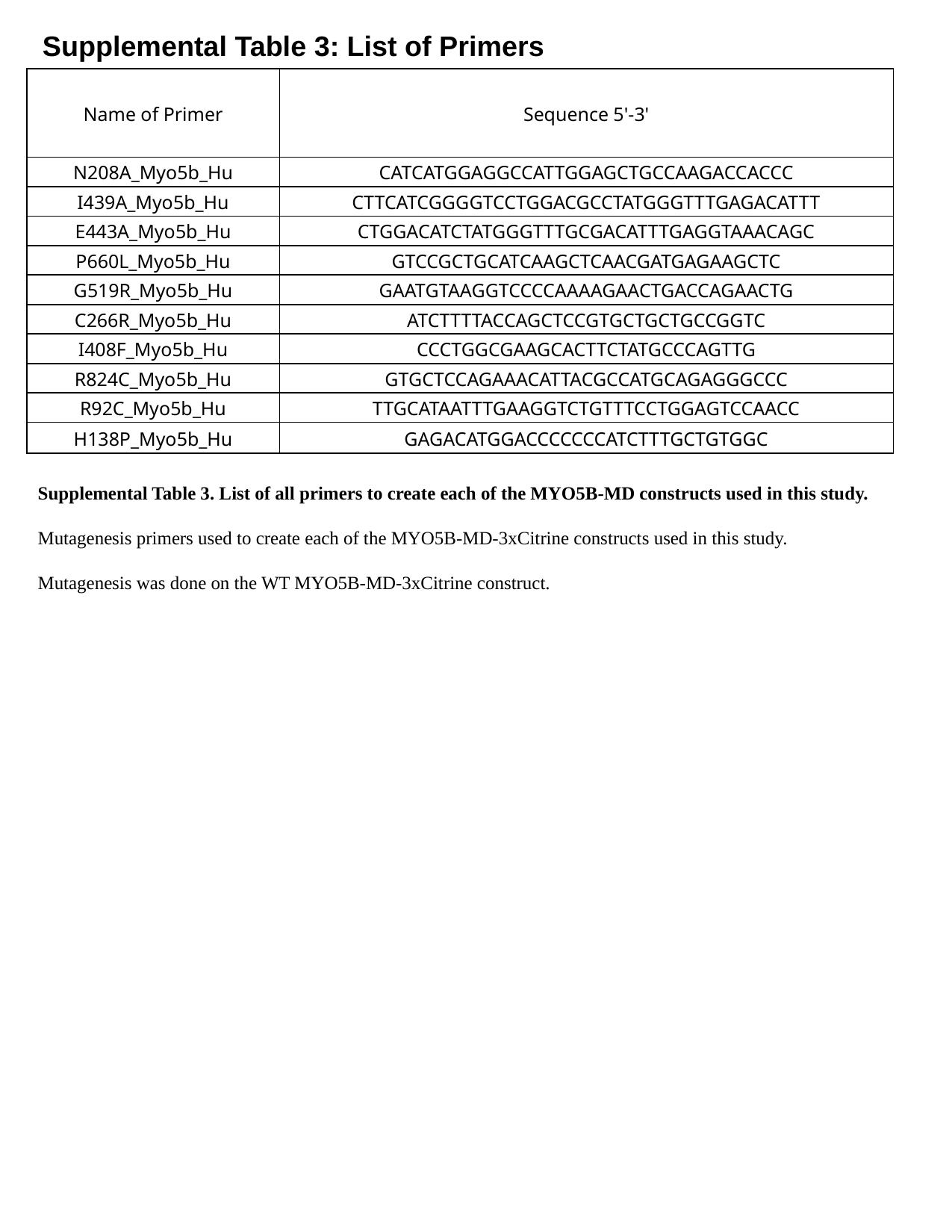

Supplemental Table 3: List of Primers
| Name of Primer | Sequence 5'-3' |
| --- | --- |
| N208A\_Myo5b\_Hu | CATCATGGAGGCCATTGGAGCTGCCAAGACCACCC |
| I439A\_Myo5b\_Hu | CTTCATCGGGGTCCTGGACGCCTATGGGTTTGAGACATTT |
| E443A\_Myo5b\_Hu | CTGGACATCTATGGGTTTGCGACATTTGAGGTAAACAGC |
| P660L\_Myo5b\_Hu | GTCCGCTGCATCAAGCTCAACGATGAGAAGCTC |
| G519R\_Myo5b\_Hu | GAATGTAAGGTCCCCAAAAGAACTGACCAGAACTG |
| C266R\_Myo5b\_Hu | ATCTTTTACCAGCTCCGTGCTGCTGCCGGTC |
| I408F\_Myo5b\_Hu | CCCTGGCGAAGCACTTCTATGCCCAGTTG |
| R824C\_Myo5b\_Hu | GTGCTCCAGAAACATTACGCCATGCAGAGGGCCC |
| R92C\_Myo5b\_Hu | TTGCATAATTTGAAGGTCTGTTTCCTGGAGTCCAACC |
| H138P\_Myo5b\_Hu | GAGACATGGACCCCCCCATCTTTGCTGTGGC |
Supplemental Table 3. List of all primers to create each of the MYO5B-MD constructs used in this study. Mutagenesis primers used to create each of the MYO5B-MD-3xCitrine constructs used in this study. Mutagenesis was done on the WT MYO5B-MD-3xCitrine construct.

## Slide 6
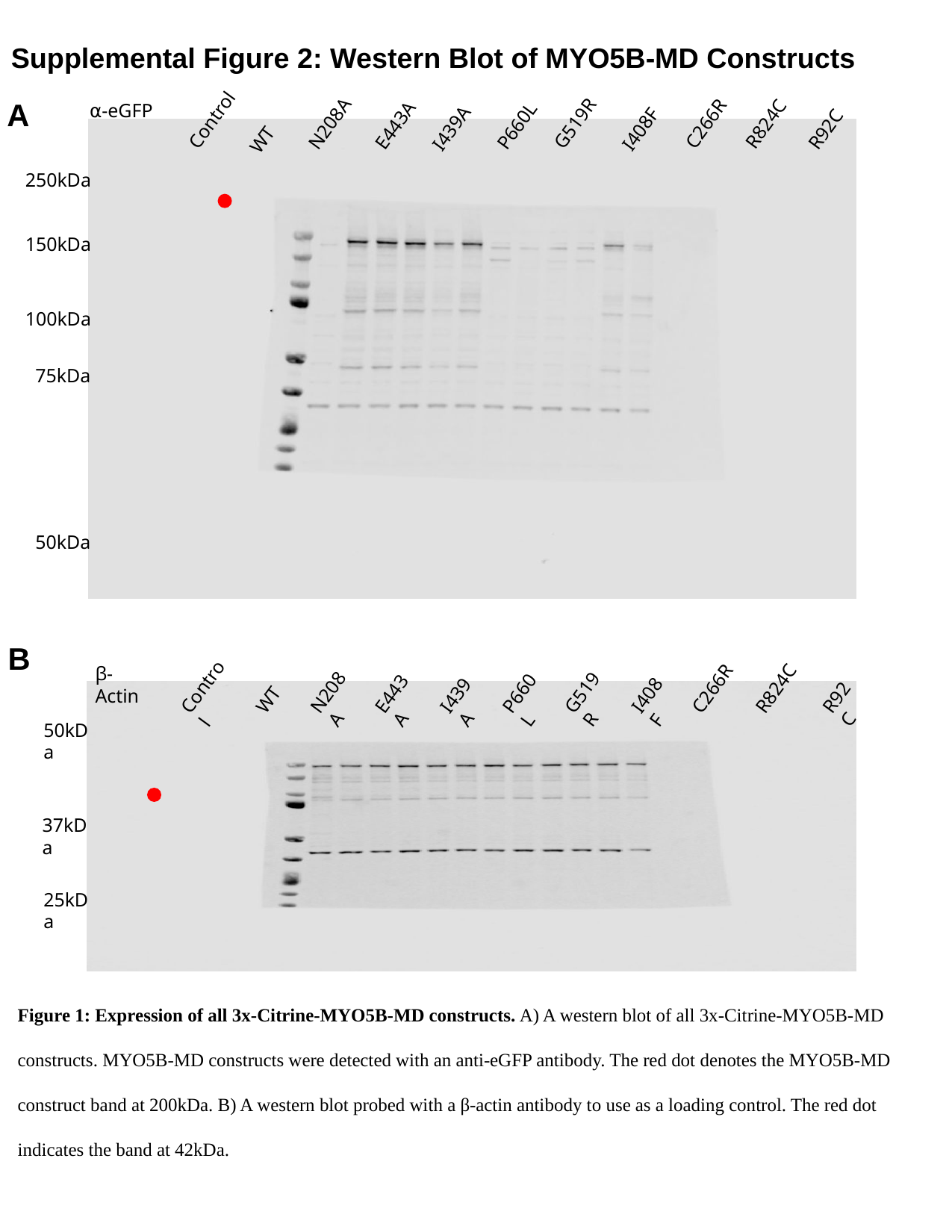

Supplemental Figure 2: Western Blot of MYO5B-MD Constructs
⍺-eGFP
Control
G519R
N208A
C266R
R824C
E443A
P660L
I439A
I408F
R92C
WT
250kDa
150kDa
100kDa
75kDa
50kDa
A
B
β-Actin
Control
WT
G519R
N208A
C266R
R824C
E443A
P660L
I439A
I408F
R92C
50kDa
37kDa
25kDa
Figure 1: Expression of all 3x-Citrine-MYO5B-MD constructs. A) A western blot of all 3x-Citrine-MYO5B-MD constructs. MYO5B-MD constructs were detected with an anti-eGFP antibody. The red dot denotes the MYO5B-MD construct band at 200kDa. B) A western blot probed with a β-actin antibody to use as a loading control. The red dot indicates the band at 42kDa.
